# Lack of benefits for prevention of cardiovascular disease with aspirin therapy in type 2 diabetic patients - a longitudinal observational study

**DOI:** 10.1186/1475-2840-8-57

**Published:** 2009-10-30

**Authors:** Wilson Y Leung, Wing-yee So, Derek Stewart, Augustine Lui, Peter C Tong, Gary T Ko, Alice P Kong, Ronald C Ma, Francis K Chan, Xilin Yang, Sau-chu Chiang, Juliana C Chan

**Affiliations:** 1 Department of Medicine and Therapeutics, The Chinese University of Hong Kong, Hong Kong SAR, PR China; 2 School of Pharmacy, Faculty of Health and Social Care, The Robert Gordon University, Aberdeen, UK; 3 Chief Pharmacy Office, Hospital Authority, Hong Kong SAR, PR China; 4 Hong Kong Institute of Diabetes and Obesity, The Chinese University of Hong Kong, Hong Kong SAR, PR China

## Abstract

**Background:**

The risk-benefit ratio of aspirin therapy in prevention of cardiovascular disease (CVD) remains contentious, especially in type 2 diabetes. This study examined the benefit and harm of low-dose aspirin (daily dose < 300 mg) in patients with type 2 diabetes.

**Methods:**

This is a longitudinal observational study with primary and secondary prevention cohorts based on history of CVD at enrolment. We compared the occurrence of primary composite (non-fatal myocardial infarction or stroke and vascular death) and secondary endpoints (upper GI bleeding and haemorrhagic stroke) between aspirin users and non-users between January 1995 and July 2005.

**Results:**

Of the 6,454 patients (mean follow-up: median [IQR]: 4.7 [4.4] years), usage of aspirin was 18% (n = 1,034) in the primary prevention cohort (n = 5731) and 81% (n = 585) in the secondary prevention cohort (n = 723). After adjustment for covariates, in the primary prevention cohort, aspirin use was associated with a hazard-ratio of 2.07 (95% CI: 1.66, 2.59, p < 0.001) for primary endpoint. There was no difference in CVD event rate in the secondary prevention cohort. Overall, aspirin use was associated with a hazard-ratio of 2.2 (1.53, 3.15, p < 0.001) of GI bleeding and 1.71 (1.00, 2.95, p = 0.051) of haemorrhagic stroke. The absolute risk of aspirin-related GI bleeding was 10.7 events per 1,000 person-years of treatment.

**Conclusion:**

In Chinese type 2 diabetic patients, low dose aspirin was associated with a paradoxical increase in CVD risk in primary prevention and did not confer benefits in secondary prevention. In addition, the risk of GI bleeding in aspirin users was rather high.

## Background

Type 2 diabetes is associated with 2-3 fold increased risk of cardiovascular disease (CVD) and is a leading cause of mortality and morbidity [1,2]. In a meta-analysis consisting of Caucasian populations, aspirin therapy reduced the combined risk of myocardial infarction by 28% but had no effect on total mortality and stroke [3]. Despite the high risks for CVD in type 2 diabetes, most of the data relating to the use of aspirin and anti-platelet agents in diabetes come from post-hoc analysis of large clinical trials with small numbers and insufficient statistical power [4]. In a recent multicentre, placebo-controlled, randomized trial, aspirin treatment with or without anti-oxidant did not confer beneficial effects in diabetic patients who had no clinical evidence of CVD [5].

In both primary and secondary prevention trials, aspirin use is associated with a 2-fold increased risk for gastrointestinal (GI) bleeding and 80% increased risk of haemorrhagic stroke [4,6]. Besides, studies from Asian populations suggest high rates of GI bleeding in patients receiving aspirin (38.5% patients on aspirin had gastroduodenal mucosal injury) [7]. This may in part explain the relatively low rate of aspirin usage in Asian patients with CVD or associated risk factors [8]. Other researchers have reported higher rates of haemorrhagic stroke in Chinese than their Caucasian counterparts (23-52% vs. 9-18% of all strokes) [9,10]. In the CHARISMA Study which compared dual therapy of clopidogrel plus aspirin versus placebo plus aspirin, a higher rate of primary end point (fatal or non-fatal CVD) was observed in patients with multiple risk factors including diabetes treated with dual therapy (6.6%) than those treated with aspirin alone (5.5%) [11].

In light of the uncertain clinical effects of aspirin therapy and relative paucity of data in diabetic patients, we undertook a prospective analysis to examine the benefit (avoidance of vascular events) and harm (occurrence of major bleeding) of low-dose aspirin therapy (75 to 325 mg per day) for primary and secondary prevention of CVD in Chinese Type 2 diabetic patients.

## Methods

### Subjects

Type 2 diabetic patients aged 30 years or above were selected from the Hong Kong Diabetes Registry established since 1995. Using an electronic prescription database, we identified patients who had received low-dose aspirin (75-325 mg per day) [5] during the observational period ("aspirin users") and those who had not ("non-users"). For each subject, the index (baseline) date referred to the date of enrolment to the Registry; or the date of aspirin initiation if patients were started on aspirin after the diabetes assessment. This avoided the error of giving the aspirin users "immortal" person-time (i.e. the error in overestimating the time to development of endpoint if the index date taken was earlier than the time when aspirin was initiated) [12]. History of GI bleeding was confirmed using databases of hospital admission and endoscopy reports, dated back to 1995. Exclusion criteria included known history of malignancy, haemorrhagic stroke, upper GI bleeding, or any medical condition predisposing to GI bleeding, including alcoholism, oesophageal varices, Mallory-Weiss syndrome, liver cirrhosis or coagulopathies [13], as recorded in the endoscopy reports. The exclusion criteria were identified from discharge diagnosis based on International Classification of Diseases, Ninth Revision (ICD-9) codes, except for upper GI bleeding for which endoscopy reports were used to improve the sensitivity of event detection. The analysis of the Registry was approved by the Clinical Research Ethics Committee, the Chinese University of Hong Kong and complied with the Declaration of Helsinki. All patients, upon enrolment to the Registry, provided written informed consent for data analysis and research purpose.

### Hong Kong Diabetes Registry

The Hong Kong Diabetes Registry was established in 1995 for quality assurance and improvement purposes at the Prince of Wales Hospital [14], which is a regional hospital serving a 1.2 million population. All patients undergo a 4-hour assessment modified from the European DIABCARE protocol [15]. Once a diabetic subject had undergone the comprehensive assessment, he/she was considered to have entered this study cohort and would be followed up till death.

Due to the heavily subsidized hospital care and less well developed primary care system in Hong Kong, the majority of patients with chronic diseases such as diabetes are managed in public hospital clinics where medications are dispensed on site. Since December 1996, all medication history, laboratory results and discharge summaries had been computerized and are available at the Hong Kong Hospital Authority (HA) Headquarter which is the governing body of 41 public hospitals which account for 95% hospital beds.

### Primary and secondary prevention

The study population was stratified into the primary and secondary prevention cohorts. The former was defined as patients with no known history of occlusive vascular disease at enrolment. These included coronary heart disease (CHD) defined as angina with positive stress test or imaging, hospitalization with myocardial infarct (MI), stroke, transient ischaemic attack (TIA), or peripheral vascular disease (PVD) defined as history of lower extremity amputation, absent foot pulses confirmed by ankle:brachial ratio less than 0.9 on Doppler ultrasound scan. The secondary prevention cohort was defined as patients with known history of one or more of the above predefined vascular diseases at baseline. Patients treated with nitrate at baseline were also included in the secondary prevention cohort [16].

### Clinical endpoints

Follow-up time was calculated as the period from enrolment to the first CHD event, death or 30^th ^July 2005, whichever came first. Clinical endpoints were ascertained using the computerised records of endoscopy, hospital discharges and death as retrieved from the HA Central Computer System. Primary and secondary endpoints were defined to examine the potential benefit (in reducing major vascular events) and harm (in causing upper GI bleeding and haemorrhagic stroke) associated with aspirin use. The "primary composite endpoint" was defined as a combination of death from a vascular cause and major vascular events, including hospitalizations due to non-fatal MI and/or non-fatal stroke, whichever occurred earlier. Vascular causes included cardiac, cerebrovascular, venous thromboembolic, haemorrhagic, and other vascular causes, in accordance with the meta-analysis by the Antithrombotic Trialists' Collaboration [17].

"Secondary endpoints" included upper GI bleeding and haemorrhagic stroke. Since upper GI bleeding is a non-specific diagnosis which can be due to oesophagitis, varices, and malignancy in addition to gastroduodenal ulcer, we defined ulcer bleeding based on the endoscopy reports which are computerized in all HA hospitals. Hence, ulcer bleeding was defined as gastric or duodenal ulcer presented as haematemesis, melena, or coffee ground vomiting as documented in the upper GI endoscopy reports by gastroenterologist [18]. All patients admitted with suspected stroke underwent computer tomography (CT) scan imaging to distinguish haemorrhagic from ischaemic stroke according to HA clinical guidelines [19].

The occurrence of vascular endpoints was identified from the principal discharge diagnoses using the ICD-9 codes including fatal or non-fatal CHD (codes 410-414); congestive heart failure (code 428), all stroke [fatal or non-fatal] (codes 430-438), haemorrhagic stroke (codes 430-432), ischaemic stroke (codes 433-435) or coronary revascularization (codes 36.0-36.1). CHD was defined as MI (code 410) or ischemic heart disease (code 411-414).

### Sample size estimation

In a pilot analysis of 5,000 patients enrolled in the Hong Kong Diabetes Registry [20], amongst aspirin non-users, the primary annual CVD event rate was 2.5% and 10% amongst those with prior history of CVD. For a 10-year observational study and assuming a 25% risk reduction with aspirin [5], 5,000 patients would be required to achieve 80% power at a 5% two-sided, alpha level based on the proportional hazards model for the primary prevention analysis and 900 patients for secondary prevention analysis.

### Statistical analysis

Analyses were performed using the Statistical Package for Social Sciences (SPSS, version 14.0) software. All data are expressed as mean ± standard deviation (SD), median (interquartile range IQR) or geometric mean (95% confidence interval [CI]) as appropriate. Serum triglyceride (TG) and creatinine, and spot urine albumin-creatinine ratio (ACR) were logarithmically transformed due to skewed distributions. Chi-square (χ^2^) test and Student's t-test were used for between-group comparisons, as appropriate. Cox proportional hazards models were used to calculate the hazard ratios (HR) with 95% CI for the primary and secondary endpoints associated with aspirin use. For each of the outcome measures, Kaplan-Meier procedure was used to estimate the survival curves for aspirin users and non-users. The proportional hazards assumption was checked by examining the plots of the hazard functions for each group. The primary composite endpoint and its components were analysed separately for the primary and secondary prevention cohorts.

Multivariable techniques were used to control for potential confounders including age, gender, smoking habit, alcohol intake, duration of diabetes, retinopathy, sensory neuropathy, PVD, history of CVD, body mass index, blood pressure (BP), serum lipids, glycated haemoglobin (HbA_1c_), albuminuria, serum creatinine, and usage of antihypertensive, antidiabetic, anticoagulant, and lipid-lowering drugs at baseline. For the analysis of GI bleeding, the usage of nonsteroidal anti-inflammatory drugs, corticosteroids, and acid-suppressing agents were included as covariates to adjust for potential confounding effects [21]. A p value < 0.05 (2-tailed) was considered significant.

## Results

### Demographics

**Figure 1:**
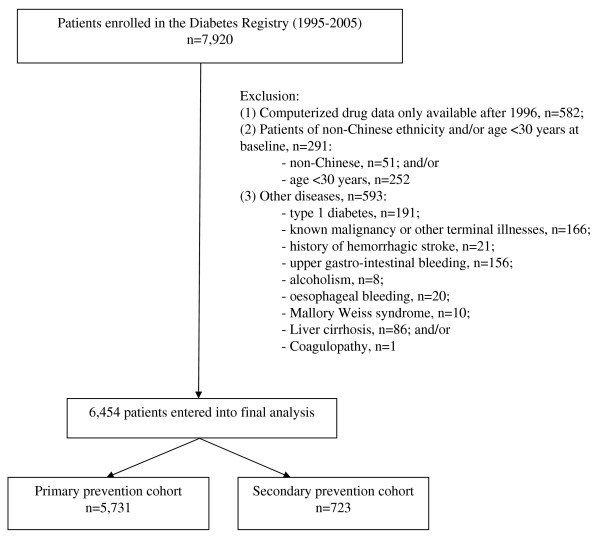
**Patients included into the final analysis according to the inclusion and exclusion criteria**.

Figure 1 summarises the patients included in the final analysis. From the original database of 6454 patients, 5731 patients (age: 58.0 ± 12.8 years; disease duration: 8.1 ± 6.9 years) entered the primary prevention cohort and 723 patients, secondary prevention cohort. The percentage of aspirin usage was 18% (n = 1034) and 81% (n = 585) in the secondary prevention cohort. The mean follow-up time from baseline to death or study end (July 2005) was 4.6 ± 2.5 years (median [IQR]: 4.7 [4.4] years), giving a total of 29,618 person-years of follow-up. In both the primary and secondary cohorts, aspirin users had more adverse cardio-metabolic risk profiles, increased usage of cardiovascular drugs, anti-diabetic drugs and histamine H_2 _receptor blockers than aspirin non-users (Tables 1, 2).

**Table 1:** Baseline clinical and biochemical characteristics between aspirin users and non-users in the complete cohort (n = 6,454)

	**Overall** **(n = 6,454)**	**Aspirin users** **(n = 1,619)**	**Non-aspirin users** **(n = 4,835)**	**p-value‡**
Age, years	58.0 ± 12.8	65.7 ± 10.2	55.4 ± 12.6	< 0.001
Male, n (%)	2,928 (45.4)	819 (50.6)	2,109 (43.6)	< 0.001
Duration of diabetes, years	8.1 ± 6.9	11.4 ± 7.5	7.0 ± 6.3	< 0.001
Smoking (current), n (%)	1,033 (16.1)	253 (15.7)	780 (16.2)	< 0.001
Alcohol (current), n (%)	517 (8.1)	100 (6.2)	417 (8.7)	< 0.001
Systolic blood pressure, mmHg	136.2 ± 20.3	142.6 ± 21.7	134.1 ± 19.4	< 0.001
Diastolic blood pressure, mmHg	75.1 ± 10.6	75.1 ± 11.2	75.1 ± 10.3	0.848
Body weight, kg	63.4 ± 12.3	63.3 ± 11.8	63.5 ± 12.5	0.583
Body mass index, kg/m^2^	25.2 ± 3.9	25.3 ± 3.7	25.2 ± 4.0	0.135
Waist circumference (cm): Males	88.7 ± 9.6	90.1 ± 8.7	88.4 ± 9.8	< 0.001
Females	83.8 ± 10.0	85.5 ± 9.9	83.6 ± 9.9	< 0.001
Glycosylated haemoglobin, %	7.59 ± 1.69	7.72 ± 1.68	7.57 ± 1.69	< 0.001
Fasting plasma glucose, mmol/L	8.59 ± 3.10	8.57 ± 3.18	8.59 ± 3.09	0.046
Total cholesterol, mmol/L	5.21 ± 1.05	5.09 ± 1.06	5.23 ± 1.05	0.167
LDL cholesterol, mmol/L	3.19 ± 0.95	3.06 ± 0.97	3.21 ± 0.94	0.286
HDL cholesterol, mmol/L	1.30 ± 0.38	1.26 ± 0.33	1.31 ± 0.39	< 0.001
Serum triglycerides, mmol/L †	1.45 (1.43, 1.47)	1.58 (1.53, 1.64)	1.43 (1.41, 1.45)	< 0.001
Serum creatinine, μmol/L †	83.2 (82.4, 83.9)	97.1 (95.0, 99.2)	79.1 (78.3, 79.8)	< 0.001
Estimated GFR^#^	74.6 (73.8, 75.3)	58.6 (56.8, 60.5)	77.6 (76.8, 78.5)	< 0.001
Spot urine ACR, mg/mmol †	3.7 (3.6, 3.9)	8.3 (7.4, 9.2)	2.9 (2.7, 3.0)	< 0.001
Blood haemoglobin, g/dL	13.7 ± 1.6	13.4 ± 1.7	13.8 ± 1.6	< 0.001
Peripheral vascular disease, n (%)	400 (6.2)	223 (13.8)	177 (3.7)	< 0.001
Lower extremity amputation, n (%)	26 (0.4)	13 (0.8)	13 (0.3)	0.003
Ischaemic heart disease, n (%)	281 (4.4)	240 (14.8)	41 (0.8)	< 0.001
Myocardial infarction, n (%)	67 (1)	65 (4)	2 (0)	< 0.001
Coronary revascularisation, n (%)	77 (1.2)	75 (4.6)	2 (0)	< 0.001
Congestive heart failure, n (%)	119 (1.8)	85 (5.3)	34 (0.7)	< 0.001
Stroke, n (%)	256 (4)	221 (13.7)	35 (0.7)	< 0.001
Atrial fibrillation, n (%)	70 (1.1)	35 (2.2)	35 (0.7)	< 0.001

Mean ± SD or number of patients (%) or † geometric mean (95% confidence interval).

‡ By Chi-square test for categorical variables and *t*-test for continuous variables

^#^Based on the abbreviated MDRD (Modification of Diet in Renal Disease) equation for Chinese (ref. 42)

LDL and HDL = low- and high-density lipoprotein, ACR = albumin-to-creatinine ratio, GFR = glomerular filtration rate.

**Table 2:** Baseline patterns of drug usage between users and non-users of aspirin in the complete cohort (n = 6,454).

	**Overall** **(n = 6,454)**	**Aspirin users** **(n = 1,619)**	**Non-aspirin users** **(n = 4,835)**	**p-value†**
**Anti-diabetic drugs**				
Insulin	1,203 (18.6%)	458 (28.3%)	745 (15.4%)	< 0.001
Any oral agent	4,301 (66.6%)	1,128 (69.7%)	3,173 (65.6%)	0.003
Sulphonylurea	3,461 (53.6%)	928 (57.3%)	2,533 (52.4%)	0.001
Metformin	3,189 (49.4%)	839 (51.8%)	2,350 (48.6%)	0.025
Thiazolidinedione	27 (0.4%)	9 (0.6%)	18 (0.4%)	0.322

**Anti-hypertensive drugs**				
Any anti-hypertensive drugs	3,103 (48.1%)	1,135 (70.1%)	1,968 (40.7%)	< 0.001
Number of anti-hypertensive drugs*	0.8 ± 1.0	1.3 ± 1.1	0.6 ± 0.9	< 0.001
Any RAS inhibitor	1,632 (25.3%)	615 (38%)	1,017 (21%)	< 0.001
ACE inhibitor	1,519 (23.5%)	564 (34.8%)	955 (19.8%)	< 0.001
AII antagonist	123 (1.9%)	57 (3.5%)	66 (1.4%)	< 0.001
Thiazide or related diuretic	162 (2.5%)	61 (3.8%)	101 (2.1%)	< 0.001
Loop diuretic	217 (3.4%)	129 (8%)	88 (1.8%)	< 0.001
Beta-blocker	756 (11.7%)	386 (23.8%)	370 (7.7%)	< 0.001
Alpha-blocker	189 (2.9%)	83 (5.1%)	106 (2.2%)	< 0.001
Calcium channel blocker	1,838 (28.5%)	697 (43.1%)	1,141 (23.6%)	< 0.001
Centrally-acting agent	309 (4.8%)	117 (7.2%)	192 (4%)	< 0.001
Vasodilator	19 (0.3%)	12 (0.7%)	7 (0.1%)	< 0.001

**Other cardiovascular drugs**				
Statin	902 (14%)	434 (26.8%)	468 (9.7%)	< 0.001
Fibrate	232 (3.6%)	76 (4.7%)	156 (3.2%)	0.006
Digoxin	65 (1%)	33 (2%)	32 (0.7%)	< 0.001
Anti-arrhythmic drug	20 (0.3%)	9 (0.6%)	11 (0.2%)	0.040
Oral anticoagulant	59 (0.9%)	9 (0.6%)	50 (1%)	0.080
Antiplatelet drug other than aspirin	24 (0.4%)	14 (0.9%)	10 (0.2%)	< 0.001

**Other drugs**				
NSAID	157 (2.4%)	46 (2.8%)	111 (2.3%)	0.218
Oral corticosteroid	83 (1.3%)	19 (1.2%)	64 (1.3%)	0.643
Proton pump inhibitor	25 (0.4%)	10 (0.6%)	15 (0.3%)	0.085
Histamine H_2_-receptor antagonist	191 (3%)	87 (5.4%)	104 (2.2%)	< 0.001
Misoprostol	1 (0%)	0 (0%)	1 (0%)	0.563

Data are number of patients (%) or mean ± SD

† By Chi-square test for categorical variables and *t*-test for continuous variables

RAS = renin-angiotensin system, ACE = angiotensin-converting enzyme, AII = angiotensin II, NSAID = non-steroidal anti-inflammatory drug, RAS inhibitor = either an ACE inhibitor or AII antagonist

### Clinical endpoint of vascular outcomes and major gastrointestinal bleeding

In the primary prevention cohort, the primary composite endpoint occurred more frequently in aspirin users (13.3%, n = 138) than in non-users (5.5%, n = 260) (Table 3). Vascular death also occurred more frequently in aspirin users (5.2%, n = 54) than in non-users (1.3%, n = 61). A similar trend was observed for non-fatal MI (aspirin users, 2.6%, n = 27; non-users, 0.9%, n = 40) and non-fatal stroke (users, 6.9%, n = 71; non-users, 3.8%, n = 180). In the secondary prevention cohort, there were 145 primary composite endpoint with 116 (19.8%) events in users and 29 (21%) in non-users of aspirin. Vascular death occurred in 55 (9.4%) aspirin users and 14 (10.1%) non-users. Non-fatal MI occurred in 20 (3.4%) aspirin users and 3 (2.2%) non-users. Non-fatal stroke occurred in 55 (9.4%) aspirin users and 18 (13%) non-users. The event rates were higher in non-users except for non-fatal MI, albeit not significant.

**Table 3:** Event rates and adjusted hazard ratio (95% confidence interval, CI) for clinical endpoints using multivariable Cox regression analysis in 6454 type 2 diabetic patients stratified by use of aspirin for primary or secondary prevention of cardiovascular disease.

	**Overall**	**Aspirin users**	**Non-aspirin users**	Hazard ratio* (95% CI)	**p-value†**
**Primary prevention cohort:**	n = 5,731	n = 1,034	n = 4,697	-	**-**
Primary composite endpoint	398 (6.9%)	138 (13.3%)	260 (5.5%)	2.07 (1.66 to 2.59)	< 0.001
Vascular death	115 (2%)	54 (5.2%)	61 (1.3%)	2.61 (1.70 to 4.01)	< 0.001
Non-fatal myocardial infarct	67 (1.2%)	27 (2.6%)	40 (0.9%)	2.05 (1.11 to 3.79)	0.023
Non-fatal stroke	251 (4.4%)	71 (6.9%)	180 (3.8%)	1.52 (1.14 to 2.04)^#^	0.005^#^

**Secondary prevention cohort:**	n = 723	n = 585	n = 138	-	-
Primary composite endpoint	145 (20.1%)	116 (19.8%)	29 (21%)	0.91 (0.60 to 1.37)^#^	NS
Vascular death	69 (9.5%)	55 (9.4%)	14 (10.1%)	0.92 (0.51 to 1.69)^#^	NS
Non-fatal myocardial infarct	23 (3.2%)	20 (3.4%)	3 (2.2%)	1.42 (0.42 to 4.85)^#^	NS
Non-fatal stroke	73 (10.1%)	55 (9.4%)	18 (13%)	0.71 (0.42 to 1.23)^#^	NS

**Complete cohort:**	n = 6,454	n = 1,619	n = 4,835	-	-
Upper gastrointestinal bleeding	138 (2.1%)	70 (4.3%)	68 (1.4%)	2.19 (1.53 to 3.15)	< 0.001
Endoscopically-confirmed ulcer bleeding	68 (1.1%)	33 (2%)	35 (0.7%)	1.72 (1.02 to 2.91)	0.043
Haemorrhagic stroke	61 (0.9%)	25 (1.5%)	36 (0.7%)	1.71 (1.00 to 2.95)^#^	0.051^#^

Data are number (%) of patients

Primary composite endpoint was defined as the composite of vascular death, non-fatal myocardial infarct or non-fatal stroke in accordance with the definition adopted by the Antiplatelet Trialists' Collaboration. Endpoints were identified from the principal diagnosis and principal procedure using the ICD-9 codes.

*Multivariable Cox proportional hazards model with age, gender, smoking, alcohol, duration of diabetes, retinopathy, sensory neuropathy, peripheral vascular disease, cardiovascular history, body mass index, blood pressure, serum lipids, HbA_1c_, albuminuria, serum creatinine, and baseline usage of antihypertensive, antidiabetic, anticoagulant, and lipid-lowering drugs adjusted as covariates.

^#^Hazard ratios and p-values were from univariate analysis.

**Figure 2:**
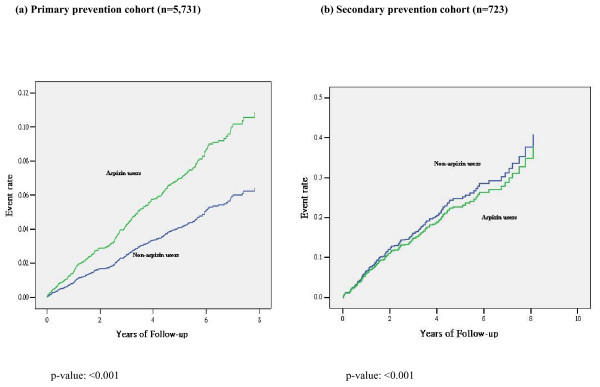
**Adjusted Kaplan-Meier curves for the primary composite endpoint (vascular death, non-fatal myocardial infarction and non-fatal stroke) in the primary and secondary prevention cohorts, stratified by aspirin usage during the observational period**.

**Figure 3:**
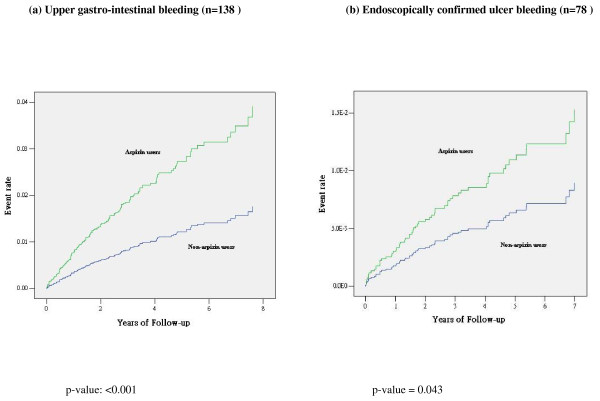
**Adjusted Kaplan-Meier curves for upper gastro-intestinal bleeding and endoscopically confirmed ulcer bleeding in the whole cohort of type 2 diabetic patients (n = 6,454), stratified by aspirin usage at baseline or during observational period**.

On multivariable analysis, aspirin use was associated with a 2-fold increased risk for the primary endpoint in the primary cohort (Table 3). The significance persisted with subgroup analysis by gender: HR (95% CI) = 1.86 (1.36, 2.54), men (n = 2560); 2.32 (1.70, 3.17), women (n = 3171) and 2.07 (1.66, 2.59), all (n = 5731). There were significant risk associations of aspirin with vascular death and non-fatal MI but not non-fatal stroke (Table 3). In the secondary prevention cohort, these risk associations were not observed (Figure 2 and Table 3). The primary composite endpoint in secondary prevention cohort remained insignificant after adjustment of gender. In the entire cohort of 6,454 patients, the rates of upper GI bleeding and endoscopically-confirmed ulcer bleeding were higher in aspirin users than non-users (Figure 3). There was also a trend for increased risk of haemorrhagic stroke in aspirin users than non-users, albeit not significant.

## Discussion

Cardiovascular events are major causes of mortality and morbidity in type 2 diabetes. Using this comprehensive Diabetes Registry, we have reported an annual CVD event rate averaging 2-3% increasing to 10% in those with cardio-renal complications at enrolment [14]. In the present analysis, the overall aspirin usage was 18% in the primary, and 80% in the secondary prevention cohort. During a 5-year observational period, aspirin use was associated with increased risk of CVD events and related death in the primary prevention cohort. In the secondary prevention cohort, similar event rates were observed between aspirin users and non-users. In the entire cohort, there was increased risk of hemorrhagic stroke and GI bleeding in the aspirin users.

Clinical guidelines in the United State recommend aspirin use for secondary prevention in type 2 diabetic patients with history of CVD and for primary prevention in those with a 10-year CHD risk of 15% or more [22]. However, these recommendations were not supported by evidence since most of the data in diabetic patients came from post-hoc analysis of large trials with marked heterogeneity [5,17,23,24]. In the meta-analysis by the Antiplatelet Trialists' Collaboration, only 6.5% of 68,814 study subjects with history of CVD had diabetes [5]. In primary prevention trials, the proportion of diabetic subjects was only 2-17% [23,24]. In a recent multicentre, randomised, placebo-controlled study with 1276 type 1 or type 2 diabetic patients who had no clinical evidence of CHD or PVD, aspirin treatment with or without anti-oxidants did not confer benefits on death or CVD event rates [5].

In our study, apart from lack of benefits, we observed a paradoxical increase in vascular risk amongst aspirin users in the primary prevention cohort. These findings are in agreement with current literature. In a subgroup analysis of the Antiplatelet Trialists' Collaboration which included data from diabetic patients (n = 1,365), the CVD event rates was 4.9% in aspirin users and 4.4% in aspirin non-users [5]. In our analysis, the non-fatal MI and stroke rates were 2.6% and 6.9% in aspirin users and the respective rates were 0.9% and 3.8% in aspirin non-users. In the Collaborative Group of the Primary Prevention Project which has the highest proportions of aspirin-treated diabetic patients [23,24], aspirin was associated with a non-significant increase in CVD deaths (RR [95% CI]: 1.23 [0.69 to 2.19]) in diabetic patients, as compared to a significant 68% reduction in aspirin-treated, non-diabetic subjects [25].

In our secondary prevention cohort, aspirin use was associated with a non-significant 9% reduction in vascular events. This lack of significance may be due to insufficient sample size (n = 723), when 900 patients are required to give the analysis an 80% statistical power. However, the very similar event rates between aspirin users (22.2%) and non-users (25.4%) strongly suggest a lack of benefit of aspirin. In the first meta-analysis performed in 1994 by the Antiplatelet Trialists' Collaboration involving 4502 diabetic patients and 42,323 non-diabetic subjects in the secondary prevention cohort, anti-platelet therapy modestly reduced the risk of vascular events by 3.8% in both groups (from 22.3% to 18.5% in diabetic group and from 16.4% to 12.6% in non-diabetic group, p < 0.001 in both groups) (5). In 2002, the same group performed a second meta-analysis of a larger cohort of 135,000 subjects. In a sub-group analysis of 9 trials which included 4,961 diabetic patients, antiplatelet therapy was associated with a non-significant 7% reduction in serious vascular events [17].

### Aspirin and bleeding complications

Aspirin therapy is associated with elevated risks of major bleeding. Aspirin may also increase risk of haemorrhagic stroke [26], which carries poor prognosis [27]. Several lines of evidence suggest that Asian including Chinese patients have higher prevalence of GI bleeding and haemorrhagic stroke than their Caucasian counterparts. In a hospital-based survey involving Hong Kong Chinese, GI bleeding accounted for 37% of all adverse drug reactions leading to admissions [28] compared to 6.0-14.5% reported elsewhere [29]. In Mainland China, a stroke surveillance program also reported a higher age-adjusted stroke incidence (Chinese vs. Caucasians: 480-800 vs. 440-650 per 100,000 person-years) and intracerebral haemorrhage (160-450 vs. 40-120 per 100,000 person-years) in Chinese aged ≥ 55 years as compared to Caucasians [30].

In our cohort, usage of low-dose aspirin was associated with a 2.2-fold increased risk of upper GI bleeding. When we restricted the analyses to endoscopically-confirmed ulcer bleeding, we still observed an adjusted 72% increased risk amongst aspirin users. Other researchers have reported 1.5-2.6 increased risk of upper GI bleeding or perforation with aspirin use at daily dosage less than 300 mg [4,13]. It remains to be proven whether this high rate of GI bleeding might have confounded the increased risk of CVD in the primary prevention cohort and that treatment non-compliance may have contributed to the lack of benefits of aspirin in secondary prevention. In our limited analysis, use of other drugs such as NSAID was not found to be associated with clinical events though the observational nature of this study precludes any conclusion.

Apart from GI bleed, there was a non-significant 70% increased risk of haemorrhagic stroke amongst aspirin users (p = 0.051) probably due to insufficient sample size. This is similar to a reported risk of 84% in a meta-analysis of low dose aspirin (average daily dosage of 273 mg) in Caucasian patients [6]. The absolute risk of haemorrhagic stroke associated with aspirin appeared to be higher in Chinese than Caucasians (3.2 vs. 1.2 additional events per 1,000 person-years treated) [6].

### Limitations

Our results need to be interpreted with caution. Firstly, our study did not distinguish between patients who received plain or enteric-coated aspirin although most studies suggested similar risks of GI bleed with different aspirin formulations [13,31,32]. Secondly, in agreement with most studies [13], we combined both cohorts in our analysis due to their similar risks of GI bleeding. Thirdly, there are considerable variations in the definitions of upper GI bleeding which may confound the risk estimation although we have used endoscopy reports to identify cases of ulcer bleeding.

While our overall results are in line with the literature, there is inevitable selection bias inherent with observational studies. Nevertheless, our comprehensive database has probably provided the largest unselected cohort of diabetic patients with relatively long duration of follow-up to address this unresolved therapeutic question. The detailed clinical and drug information including hospitalization and endoscopy reports have enabled us to adjust for confounding factors to draw important conclusions which have implications on clinical management. Our findings, which are particularly relevant to Chinese, may also explain the relatively low usage of aspirin in Asians than Caucasians [8]. Although we have not systemically examined doctors' rationale of prescribing, clinical experiences with adverse effects of aspirin may have led to cautious use of this class of drug.

## Conclusion and Implications

The baseline data of our cohort showed the aspirin users had more risk factors for CVD than non-users. After statistical analysis with adjustment of these variables, aspirin use, among Chinese type 2 diabetic patients, for primary prevention was associated with 74% increased risk of incident CVD events while no benefit was observed in the secondary prevention cohort. This conclusion needs prospective study to be confirmed. In addition, overall speaking, aspirin use was associated with a 2.2-fold increased risk of upper GI bleeding and a non-significant trend for increased risk of haemorrhagic stroke. These results are not incompatible with the current body of knowledge based on either meta-analyses or randomized clinical trials and call for re-evaluation of the current recommendation regarding the use of aspirin in diabetic patients. Given their multiple comorbidities and high risk for CVD, there is an urgent need to design studies to evaluate the risk-benefit equation regarding the use of aspirin and other anti-platelet agents in diabetic patients with detailed documentation of potential confounders. In this respect, silent GI bleed with aspirin therapy may lead to anaemia which is a major predictor for CVD events in Chinese type 2 diabetic patients [33]. Meanwhile, there is a need to draw up protocols to identify high risk subjects for GI bleeding (e.g. smokers and carriers of *Helicobacter Pyloris*) for co-administration of ulcer-healing drugs, to maximize benefits and minimize harm associated with aspirin therapy.

## Abbreviations

CVD: cardiovascular disease; GI: gastrointestinal; HA: Hospital Authority; CHD: coronary heart disease: MI: myocardial infarct; TIA: transient ischaemic attack; PVD: peripheral vascular disease; TG: triglyceride; ACR: albumin-creatinine ratio; HR: hazard ratios; BP: blood pressure

## Competing interests

The authors declare that they have no competing interests.

## Authors' contributions

WL and JC had full access to all of the data in the study and take responsibility for the integrity of the data and the accuracy of the data analysis. WL, WS and JC designed the study. WS, AL and SC collected the data. WL, XY and GK did the data analysis. WL and GK drafted the manuscript. DS, FC and JC critically reviewed the manuscript. AK, RM and PT gave the administrative, technical, or material support. All authors provided suggestions during the preparation of the manuscript and approved the final version submitted for publication.
